# Questioning the questionnaire: a Dutch national survey on generic patient-reported outcome measures in traumatology

**DOI:** 10.1186/s41687-025-00969-z

**Published:** 2025-12-02

**Authors:** Thymen Houwen, Jan C. van Ditshuizen, Roos J. M. Havermans, Ruth E. Geuze, Mariska A. C. de Jongh, Michael H. J. Verhofstad

**Affiliations:** 1 Trauma Research Unit Erasmus Medical Center, Department of Surgery, Erasmus MC University Rotterdam, Rotterdam, 3000 CA The Netherlands; 2 Network Emergency Care Brabant, Elisabeth-TweeSteden Ziekenhuis, Hilvarenbeekseweg 60, Tilburg, 5022 GC The Netherlands; 3 Trauma Centre Southwest-Netherlands, Erasmus MC, University Medical Center, Rotterdam, The Netherlands; 4 Department of Surgery, Elisabeth-TweeSteden Ziekenhuis, Tilburg, The Netherlands; 5 Department of Orthopedics and Trauma Surgery, Elisabeth-TweeSteden Ziekenhuis, Tilburg, The Netherlands

**Keywords:** Survey, Questionnaire, Patient reported outcome measures, PROM, Trauma

## Abstract

**Background:**

PROMs are fairly new in medical practice. In trauma surgical care in the Netherlands, it is unknown to what extend (generic) PROMs are embedded into clinical care. This study evaluates the awareness of generic PROMs in trauma and orthopedic surgeons in the Netherlands by assessing the current implementation, barriers and facilitators in usage of PROMs and willingness to assess other health domains besides the physical health domain.

**Methods:**

A purposive sampling survey was conducted in all active associates of the Dutch Trauma Society. Furthermore all medical members of the regional trauma assembly in multiple network regions were invited to complete the survey.

**Results:**

Generic questionnaires and information about physical health, mental health, social health and work status were acknowledged as useful In facilitating decisions and information about patients in trauma surgical care. Important assumed barriers of generic PROMs were the inability to intervene in health domains outside surgical treatment modalities (44%) and the negative influence on time management (43%). Yet, 55% of participants acknowledged generic information as the biggest advantage of generic PROMs, because of a wider therapeutic scope for specific health issues and better comparison of health outcomes between subgroups. Sustainable implementation of questionnaires required integration and fully automation of questionnaires in electronic patient files.

**Conclusion:**

Barriers have to be overcome for successful and sustainable implementation of (generic) PROMs in trauma surgical care. Broad support from trauma care professionals and the development of intelligent technological solutions that can seamlessly support clinical workflows are crucial to successful implementation.

**Supplementary Information:**

The online version contains supplementary material available at 10.1186/s41687-025-00969-z.

## Background

Patients admitted to a hospital due to physical trauma have a vast range of injury patterns: from isolated mono-trauma to severely and unstable polytrauma. Historically, survival has always been an outcome parameter in research of trauma patients. Implementation of modern and professional trauma showed that mortality numbers are decreasing [1–3]. As trauma systems develop, the medical view on health problems that trauma patients experience has been developing as well. Previous studies showed short-term and long-term limitations in physical health, mental health and social health after physical trauma [4–8]. Knowledge about non-fatal clinical outcomes is important to understand the impact of trauma on daily functioning. Patient-reported outcomes (PROs) might give tools and opportunities to gain insight in the needs and problems that trauma patients experience, this in order to deliver more personalized health care on different health areas [8–10].

PROM questionnaires are “patient-reported” without primary involvement of researchers or clinicians. Two categories of PROMs can be distinguished: disease -or organ specific and generic questionnaires [11]. Evidence exists that routinely monitoring PROs can result in significant progress of outcomes of patients like reduced hospitalizations and emergency care visits [12, 13]. Although PROMs have been evolving rapidly [14], implementation of PROMs in general routine practice is limited in some countries like the United Kingdom, Sweden, the United States and Australia [11]. Interestingly, every country is also adopting in different fields of medicine [15]. International evidence and practical experiences support the implementation of easy interpretable and feasible PROMs to stimulate patient-centered care [15, 16].

In the Netherlands, PROMs collection especially follow the context of disease-specific and condition-specific clinical registries, but generic parameters important to patients are generally missing [15]. A recent systematic review highlighted the potential importance of generic PROMs in relation to physical health, mental health and social health in trauma -and orthopedic care [14]. although this systematic review primarily focused on mental and social health, a substantial number of included papers addressed the physical health domain (i.e. physical function and pain) [14]. Within trauma surgery, some studies show the need of trauma patients for focusing on general health outcomes next to physical health and PROMs could add to informing healthcare professionals about broader patient-issues [17, 18]. It remains unclear to what extent generic -or injury specific PROMs are integrated into the routine clinical practices of trauma and orthopedic surgeons in the Netherlands. Moreover, the implementation of PROMs may vary across hospitals, given the relatively recent introduction of PROMs.

This study evaluates the current utilization of generic PROMs in trauma and orthopedic care in the Netherlands. Specifically we examined (1) the extent and nature of clinical and research applications of generic PROMs, (2) perceived barriers and facilitators in usage of generic PROMs, and (3) professional perspectives on their usefulness and the willingness to assess health domains beyond physical functioning.

## Methods

This voluntary Dutch survey was developed by TH, MdJ and MV and administered via a secured web-based questionnaire (SurveyMonkey™). The Cherries-checklist for reporting e-surveys was used to guide the reporting of this study [19]. A brief introduction outlining the survey’s length and scientific objectives was provided on the first page, followed by five thematic blocks of questions: (1) Six items on demographic information (e.g. years of experience, gender, professional role, affiliation and current position); (2) Five items regarding the general use of questionnaires within the respondent’s hospital; (3) Ten items assessing personal views on the relevance and type of health domains; (4) Seven items exploring perceived barriers and facilitators to the use of generic PROMs; (5) Ten items evaluating specific criteria that PROMs should meet. Most questions were multiple choice with occasional short-answer options. Personal beliefs and opinions were assessed using a six-point Likert scale (always, very frequently, frequently, occasionally, rarely, never), while specific conditions that PROs should meet were scored on a five-point Likert scale (strongly agree, agree, neither agree nor disagree, disagree, strongly disagree). Additional questions were dichotomous (‘yes’,‘no’), with the option to provide free text responses. Adaptive questioning was incorporated to reduce the number and complexity of items. For example when participants answered “no” to the question regarding the use of PROMs in their hospital, subsequent detailed questions about PROM implementation were omitted. Participants were able to review and modify their responses prior to submission. The original Dutch version of the survey is available in Supplemental File 1, an English translation is provided in Supplemental File 2.

Medical professionals from level I, II and III trauma centers in the Netherlands were invited to participate. In the Dutch trauma system, level I trauma centers manage the most severe (multi)trauma cases and complex mono trauma patients, and always include neurosurgical capabilities. Level II hospitals provide less specialized care, lack neurosurgical services, but are equipped to stabilize trauma patients. Level III hospitals are limited to treating patients with isolated mono-traumatic injuries.

The survey was reviewed and tested by a panel of health care professionals including three trauma surgeons, one orthopedic trauma surgeon, one surgical resident, one policy maker of the Network Emergency Care Brabant and one clinical epidemiologist. The survey was revised through multiple rounds of feedback until consensus was reached. The content of the survey was based on recommendations and literature reviews in the Netherlands and Australia [15, 16]. The first report focused on widely supported and comprehensible PROMs aimed at promoting patient-centered care in the adult population [16].The second report, published by the Australian Health Services Research Institute, presented a literature review on the evidence and practical experiences related to the collection and use of PROMs in healthcare settings [15].

We employed the method of purposive sampling (a form of non-probability sampling) in which all (but limited to) active associates of the Dutch Trauma Society (*n* = 350) were invited by email after approval of the questionnaire by the board of the Dutch Trauma Society [20]. The society’s secretary included an invitation text and survey link in the monthly newsletters of September 2022 and October 2022. Additionally, all medical members of the regional trauma assemblies (in Dutch: Regionaal trauma overleg) across multiple network regions- Brabant, Euregio, Limburg, Noord-Holland Flevoland, Southwest Netherlands, Utrecht and Zwolle- were invited to participate via email (*n* = 106). A reminder was sent two weeks after the initial invitation. Although duplicate invitations may have occurred, a clear instruction was provided to submit only one response. The survey was open-access and did not involve password-protection, in order to lower the threshold for participation. No financial or non-financial incentives were offered. Collected data were pseudo anonymized and could be linked to individual hospitals, but not to individual respondents.

IBM SPSS Statistics for Windows, version 24.0 (IBM Corp., Armonk, N.Y., USA) was used for descriptive statistics to report the outcomes. Results were analyzed using descriptive statistics and showed as proportions, mean ± SD or medians with ranges of distribution and 95% confidence intervals (CI).

## Results

### Responders

In total, four hundred sixty-five individuals were invited to participate in the study. Ninety-seven (21%) trauma professionals responded to the survey, of which eighty-seven completed the survey (17%), eight partly completed the survey (2%), and one did not fill out the survey at all. Especially trauma surgeons responded (*n* = 75, 78%), followed by twelve orthopedic trauma surgeons and ten either residents in training or physician assistants. Eighty-three (86%) of the participants were males and years of experience was well distributed between participants. Forty-six (48%) of the participants were working in a level I trauma center. Most participants stated that PROMs were only used in specific clinical research projects (*n* = 32, 33%) or were not used at all (*n* = 24, 25%) in daily practice. When used, especially EuroQol-5D, SF-36, Patient Reported Outcomes Measurement Information System (PROMIS®) and SF-12 were generally used by trauma surgeons or orthopedic trauma surgeons. (Table 1 for all demographic information).

**Table 1:** Demographic information of participants

Variable	*n* = 97 (100%)
Gender	
Male	83 (86%)
Female	14 (14%)
Specialty	
Trauma surgeon	75 (78%)
Orthopedic trauma surgeon	12 (12%)
Other	10 (10%)
Main Affiliation	
Academic level I trauma center	24 (25%)
Nonacademic level I trauma center	22 (23%)
Level II hospital	42 (43%)
Level III hospital	9 (9%)
Years of clinical experience	
0–5 years	24 (25%)
5–10 years	12 (12%)
10–15 years	27 (28%)
15–20 years	16 (17%)
More than 20 years	18 (19%)
Current position within hospital	
Consultant specialist and staff member	80 (82%)
Consultant specialist, but fellow	7 (7%)
Resident	8 (10%)
Other	2 (2%)

### Personal opinions on usefulness and types of (generic) PROMs

Overall, most participants supported the opinion of additional value and usefulness of generic questionnaires to the daily trauma surgical practice (occasionally *n* = 35, 40%, often *n* = 22, 25%, very often *n* = 14, 16% and always *n* = 7, 8%). In the view of shared decision-making, participants mostly saw some positive contributions of generic questionnaires (occasionally *n* = 38, 44%, often *n* = 27, 31%).

Participants of this survey emphasized the relevance within trauma surgical practice of generic information on physical health (occasionally *n* = 4, 5%, often *n* = 36, 42%, very often *n* = 26, 30%, Always *n* = 20, 23%), mental health (occasionally *n* = 24, 28%, often *n* = 38, 44%, very often *n* = 16, 19%, always *n* = 5, 6%), social health (occasionally *n* = 25, 29%, often *n* = 36, 42%, very often *n* = 15, 17%, always *n* = 7, 8% and work status (occasionally *n* = 34, 40%, often *n* = 29, 34%, very often *n* = 14, 16%, always *n* = 2, 2%).

### Barriers and facilitators in the use of (generic) PROMs in trauma surgical practice

The most frequently reported barrier to the use of generic PROMs was the collection of information that falls outside the scope of surgical practice and cannot be directly influenced or acted upon by the surgical professional (*n* = 43, 44%). This was followed by concerns regarding the potential negative impact of generic questionnaires on time-management (*n* = 42, 43%) and the perception that generic questionnaires do not add meaningful value to current clinical practice (*n* = 25, 26%).

Trauma professionals also identified potential barriers for patients in completing PROMs. The most frequently cited concern was time investment required (*n* = 69, 71%). Other notable barriers included Insufficient patient understanding of the added value of completing questionnaires (*n* = 45, 46%), the practice of administering questionnaires without discussing the results (*n* = 35, 36%), and low (digital) literacy among patients (*n* = 31, 32%).

Although also seen as a disadvantage, survey-participants identified generic health information about the patient as one of the key advantages of using generic PROMs (*n* = 53, 55%). This was followed by the potential to improve comparability of health outcomes across patient subgroups (*n* = 32, 33%) and enhanced preparation for outpatient clinic visits- both for patients (*n* = 29, 30%) and surgeons (*n* = 28, 29%). Finally, the primarily objective of using generic questionnaires, as indicated by respondents, should be to evaluate the effectiveness of interventions and treatments on health-related quality of life (*n* = 49, 51%).

## Discussion

This study surveyed the current application of generic PROMs in trauma and orthopedic health care in the Netherlands. Specifically, it assessed the extent and nature of their clinical use by examining barriers and facilitators of generic PROMs, as well as exploring clinicians’ views on the relevance of PROMs and their willingness to evaluate health domains beyond physical functioning.

Mostly male trauma surgeons (*n* = 83, 86%) working in a level I trauma center, or level II trauma facility, participated in this survey. More than half (*n* = 56, 58%) of the participants used PROMs exclusively for research purposes or did not use PROMs at all.

In general, participants expressed a positive attitude towards the additional value and practical utility of generic questionnaires in routine trauma surgical practice, particularly in supporting decision-making. Generic information about a patients‘ physical health, mental health, social health and work status was considered relevant within the context of trauma care.

In addition to these positive findings, barriers were observed as well. Inability to intervene in certain health problems outside the surgical professional scope (*n* = 43, 44%) was the most important barrier followed by the presumed negative impact on time management (*n* = 42, 43%). Moreover, more than 25% (*n* = 25) of participants indicated the lack of additional value of PROMs to the current surgical practice. This perception contrasts the previous positive attitudes toward the relevance of generic questionnaires in capturing physical, mental and social health status. The discrepancy may stem from surgeons’ limited capacity to personally intervene in domains such as mental or social health, despite recognizing their clinical relevance. Furthermore, generational differences in attitudes toward the use of PROMs may exist among trauma surgeons. It is also important to acknowledge that survey-based research cannot distinguish between socially desirable responses and genuine opinions. To further explore the current results, a follow-up qualitative study involving interviews could further elucidate trauma surgeons’ perspectives on the role of generic information in clinical practice.

In the use of generic PROMs, recommendations are provided on a national level based on recent literature [21]. A Dutch working group with representatives of umbrella organizations involved in Dutch medical care, patient organizations and PROMs experts developed a standard set of generic PROMs that could be implemented in Dutch medical care. This set includes generic PROMs related to pain, fatigue, mental functioning, physical functioning, participation in social roles, perceived health and quality of life. Additionally, the working group recommended the use of disease-specific PROMs for certain (unspecified) conditions [16]. In contrast, findings from our survey revealed uncertainties among trauma surgeons regarding the practical usefulness of generic PROMs. In a surgical profession, knowledge about mental health or social health might be important due to the effect on for example. recovery, complications or in hospital stay, but treatment of non-surgical problems must be supported by other medical professionals [22–24]. For this reason, the implementation of generic PROMs in surgery includes the development of a wide supporting (logistical) network of medical professionals. The working group emphasized discussion of results in clinic, limiting administrative patient burden and using PROMs in shared decision making while the survey showed that PROMs should not negatively interfere in time management. In this view, it is a precarious balance between different parties’ preferences [21, 25].

There are several arguments for the integration of PROMs into clinical practice. First, patients possess unique insights into their own physical, mental and social health, positioning them as the most accurate reporters of the limitations they experience. Second, PROMs facilitate patient-centered care by enabling the personalization of treatment plans based on individual patient experiences. Third, the use of PROMs can enhance awareness of improvement and stagnation throughout the recovery process, benefiting both healthcare professionals and patients. Fourth, structural use of PROMs may contribute to more efficient utilization of clinical resources, particularly when applied in a standardized manner. Studies have demonstrated that the use of PROMs does not adversely affect time management [26, 27]. Fifth, PROMs may support expectation management and foster realistic communication regarding future prospects and outcomes, especially when integrated into electronic health records [15].

Generic questionnaires may offer several advantages over disease-specific questionnaires. Notably, they allow for the assessment of health related quality of life across a broad range of health conditions, facilitating comparisons between different patient subgroups or between non-trauma populations. In trauma surgery, various fracture types and injuries are often associated with distinct classification systems and dedicated outcome measures. The use of a generic questionnaire can help reduce the proliferation of condition-specific tools, thereby promoting the use of more comprehensible questionnaires. In disease specific measures, psychometric properties such as internal consistency, reliability, measurement error, content validity, criterion validity, construct validity, and responsiveness can be highly variable and concepts explored are not always explicitly defined, whereas generic questionnaires can be selected based on the specific concept-needs [28–30]. additionally, employing a unified set of questionnaires enables the collection of comprehensive health data across multiple domains without necessitating separate questionnaires for each medical specialty. On top of this, generic questionnaires may reveal specific health issues that patients themselves may not yet recognize, but which can significantly influence the course of recovery (i.e. pain limiting physical functioning or maintain social isolation, thus restricting physical exercises or social participation) [14].

Several barriers are anticipated in the implementation of (generic) PROMs within surgical practice. Sustainable employability of generic PROMs is in need for accurate data collection [15].

In the Netherlands, a working group has recommended the use of PROMIS for routine clinical application [21]. Although increasing in the last years, evidence supporting the use of PROMIS within surgical disciplines remains limited compared to other medical specialties [14]. Second, PROM data must be presented in a clear and interpretable manner to ensure clinical utility, and preferably implemented in electronic health records [15, 31]. Furthermore, it is essential to demonstrate to clinicians that the time and effort required for PROM collection can contribute meaningfully to more comprehensive healthcare delivery with better patient satisfaction and improvement of daily functioning, such as is already seen in oncology [15, 32, 33]. The implementation of PROMs in clinical practice is expected to impact the workflow of outpatient clinics and requires the active engagement of all healthcare professionals involved. Simple, automated and low time-consuming processes involved in PROMs are important for long-term success. Questionnaires should be strategically integrated into key clinical encounters and serve a clearly defined purpose that is also communicated to patients (e.g. monitoring progress, screening tool, reference value for future measurements or outcome benchmark). Furthermore, Interpretation of scores must be straightforward, with clear guidance on their clinical implications and appropriate responses to the results. Initially, all patients should receive a standardized set of questionnaires; and however, the long-term goal is to develop a self-learning system capable of identifying individual patients’ specific needs across health domains. This would allow for the administration of only the most relevant and necessary questionnaires, thereby reducing burden while maintaining clinical relevance. The overarching aim of implementing PROMs should be to enhance patient-centered care, serving as the guiding principle for their integration into clinical workflow [15, 16].

### Strengths and limitations

This paper is the first to explore needs and barriers associated with the implementation and clinical application of generic PROMs within (orthopedic) trauma surgery in the Netherlands. Although a recent national recommendation has advocated for the use of generic PROMs in Dutch healthcare, Dutch trauma professionals were not part of this. This survey involved participants from at least two of the largest trauma regions in the Netherlands, as well as professionals affiliated with designated trauma networks. Respondents represented a broad spectrum of trauma care, including level I, level II and level III trauma centers, thereby capturing a comprehensive range of perspectives in the field. Additionally, early career trauma professionals, such as trauma surgical residents, fellows and physician assistants were included, ensuring that the views of the wider trauma care community were adequately represented.

Some limitations coexist with this study. First, the survey sample predominantly included more experienced trauma professionals, with relatively fewer responses from younger fellows or young specialists. Given the inclusive nature of PROMs, a more balanced representation across experience levels would have been preferable. Second, selection bias may have influenced the results, as a significant number of participating trauma surgeons was affiliated with a level I trauma center. Conversely, trauma surgeons in Level II and III centers may perceive less added value in PROMs and may have been less inclined to participate. Additionally, as PROMs are relatively novel and closely linked to developments in health information technology, generational differences in attitudes and familiarity may have influenced responses. Finally, although patient perspectives are essential in evaluating the implementation of PROMs, this study did not include input from a patient federation, as no dedicated organization currently exists for trauma patients in the Netherlands.

## Conclusion

Overall, this study provides an initial insight into the (future) implementation and clinical use of generic PROMs within the field of trauma surgery. The findings indicate a generally positive perception of the added value and utility of generic PROMs among trauma professionals. However, barriers must be addressed to ensure sustainable implementation. Acknowledgement for the limited availability of surgical interventions for certain health issues identified by PROMs and concerns regarding time management need to be taken into account. Achieving successful integration will require broad support from trauma care professionals, as well as the development of intelligent technological solutions that can seamlessly support clinical workflows.

## Supplementary Information

Below is the link to the electronic supplementary material.

Supplementary Material 1

Supplementary Material 2

## Data Availability

All data generated or analyzed during this study are included in this published article and its supplementary information files.
