# Supplementary material for: Questioning the questionnaire: a Dutch national survey on generic patient-reported outcome measures in traumatology
Source: J Patient Rep Outcomes. 2025 Dec 2;9:139. doi: 10.1186/s41687-025-00969-z (PMC12672991; doi:10.1186/s41687-025-00969-z)
Supplement: Supplementary file 1 — Supplementary Material 1 [file 41687_2025_969_MOESM1_ESM.docx]

Supplemental file 1. Questionnaire “generic PROMs” sent to Dutch trauma surgeons and orthopedic trauma surgeons.

**General demographic information (five questions)**

1. Years of experience as senior consultant within trauma surgery?

- 0-5 years
- 5-10 years
- 10-15 years
- 15-20 years
- More than 20 years

1. Gender?

- Male
- Female

1. What is your specialty?

- Trauma surgeon
- Orthopedic trauma surgeon
- Other, namely (free text)

1. What level is your current main affiliation?

- Academic level I trauma center
- Nonacademic level I trauma center
- Level II hospital
- Level III hospital

1. In which hospital are you currently working?

- (Free text)

1. What is your current position within the hospital?

- Consultant specialist and staff member
- Consultant specialist, but fellow
- Resident
- Other, namely (free text)

**Questions on general use of questionnaires (five questions)**

1. Are patient-reported outcomes (PROMs) being provided to trauma patients within your hospital that are initiated from your department? (Questionnaires from other specialties such as the pre-operative screening department or LROI questionnaires from the orthopedic department are not included).

- Yes, routinely condition specific questionnaires
- Yes, in selected cases condition specific questionnaires
- Yes, routinely generic questionnaires
- Yes, in selected cases generic questionnaires
- Yes, routinely condition specific questionnaires and generic questionnaires
- Yes, in selected cases condition specific questionnaires and generic questionnaires
- Yes, only study conditioned questionnaires (condition specific/ generic)
- No

1. At which moment are questionnaires are being provided to patients?

- Directly during or after presentation on the A&E department
- Before a visit of the plaster cast room
- Before a visit of the regular outpatient clinic
- Before an operation because of the injury
- After an operation
- At specifically set moments after the initial trauma (i.e. after 2 weeks, 6 weeks, 3 months)
- At specifically set moments after the operation (i.e. after 2 weeks, 6 weeks, 3 months)
- I don’t know
- Other, namely (free text)

1. In what way are questionnaires being provided to patients? (multiple answers can be given)

- By using a paper version and via a postal letter
- A fellow worker sends the questionnaire via the electronic patient file
- A fellow worker sends the questionnaire via an external program (i.e. external web viewer)
- Fully automated by the electronic patient file
- By telephone
- By using a tablet in the hospital (outpatient clinic or ward)
- By using a paper version in the hospital (outpatient clinic or ward)
- I don’t know
- Other, namely (free text)

1. In what way are questionnaires being visualized, if questionnaires are being sent by using a digital method?

- Completed questionnaires are directly visible within the electronic patient file
- Completed questionnaires are available in a web viewer which can be accessed by the electronic patient file of the individual patient
- Completed questionnaires are in a web viewer which cannot be accessed by the electronic patient file of the individual patient
- Other, namely (free text

1. Which questionnaires are being used in your department? (multiple answers are possible)

- Assessment of Quality of Life (AQoL)-8D
- European Quality Of Life-5 Dimensions (EuroQol-5D)
- Health Utility Index 2 en/of 3 (HUI2/3)
- Hospital Anxiety And Depression Score (HADS)
- Nottingham Health Profile (NHP)
- PROMIS questionnaires
- Quality of Well-Being (QWB)
- Rosser Index
- Sheehan Disability Scale (SDS)
- Short Form-6 Dimensions (SF 6D)
- Short form 12 (SF-12)
- Short form 36 (SF-36)
- Sickness Impact Profile
- World Health Organization Quality Of Life (WHOQOL-Bref)
- WHO (Five) Well-Being Index (WHO-5)
- 15D
- I don’t know
- Other, namely (free text)

**Personal opinions on usefulness and types of specific health domains (ten questions)**

1. Do you think generic patient-reported outcomes measures could provide useful information on your daily practice?

- Always
- Very frequently
- Frequently
- Occasionally
- Rarely
- Never

1. Can generic questionnaires contribute positively to the process of shared decision making in your daily practice? (i.e. in decisions about treatment options, after care or additional support)

- Always
- Very frequently
- Frequently
- Occasionally
- Rarely
- Never

1. Are you familiar with the concept of computer adaptive testing (CAT) in questionnaires?

- Yes
- No

1. CAT is based on the item-response theory. Previous questions and given answers are informing on an algorithm which selects the most suitable next question from a set of questions. Are you interested to learn more about CAT?

- Yes
- No
- Other, namely

1. Is it relevant to retrieve generic physical health information about trauma patients visiting your own department? (i.e. physical function or pain interference)

- Always
- Very frequently
- Frequently
- Occasionally
- Rarely
- Never

1. Is it relevant for treatment and the outcomes of treatment to be informed about social health issues of a patient? (i.e. social participation, social roles or social support)

- Always
- Very frequently
- Frequently
- Occasionally
- Rarely
- Never

1. Is it relevant for treatment and the outcomes of treatment to be informed about work status and performance of a patient?

- Always
- Very frequently
- Frequently
- Occasionally
- Rarely
- Never

1. Is it relevant for treatment and the outcomes of treatment to be informed about mental health issues of a patient? (i.e. anxiety, depression and anger)

- Always
- Very frequently
- Frequently
- Occasionally
- Rarely
- Never

1. Is it relevant for treatment and the outcomes of treatment to be informed about the sleeping quality of a patient?

- Always
- Very frequently
- Frequently
- Occasionally
- Rarely
- Never

1. Is it relevant for treatment and the outcomes of treatment to be informed about experienced fatigue of a patient?

- Always
- Very frequently
- Frequently
- Occasionally
- Rarely
- Never

**Barriers and facilitators (seven questions)**

1. What is the most important barrier to use generic questionnaires? Please provide a maximum of three answers.

- Different interaction between health professional and patient, because of the implementation of generic questionnaires
- The health professional has less control about the conversation, because of less specific information provided by the patient
- Not convinced of the additional value of generic questionnaires or general health information
- Some information is being provided in which the surgeon cannot directly interfere in
- Privacy aspects associated with the provision of general information
- Generic questionnaires might possibly take more time to discuss
- Other, namely (free text)

1. What will be the most important barrier to patients in completing questionnaires in general? Please provide a maximum of three answers.

- Time investment
- Privacy aspects
- Limited computer skills
- Low literacy
- Visual problems
- Insufficient insight into the additional value of questionnaires
- Providing the patient with questionnaire(s) without discussing
- Other, namely (free text)

1. As a health professional, what might be the biggest advantage to use generic questionnaires? Please provide a maximum of three answers.

- The health professional gets an insight in general health aspects of the patient
- The patient gets insight into his own general health status and the health related course
- It is easier to compare different outcomes between subgroups when the same generic questionnaires are being used.
- It is easier to compare the outcomes between patients connected to different specialties when the same generic questionnaires are being used.
- The health professional is able to prepare for questions on a broader sets of health aspects
- Outcomes can easier be used for broad scientific research purposes
- The patient will be better informed and prepared for the outpatient clinic visit
- Other, namely (free text)

1. What should be the main goal(s) in using generic questionnaires in daily practice? Please provide a maximum of three answers.

- Efficacy measurement of interventions/treatment on health-related quality of life
- Shared decision making based on generic information
- Measurement and comparison of quality parameters between colleagues
- Measurement and comparison of quality parameters between hospitals
- *Measurement of general health parameters/ general health of the patient*
- Detection of patient limitations
- Guidance in decision making
- To improve interaction between patient en health professional
- Scientific research
- Other, namely (free text)

1. If the government, health related federation or other organization would force the use of PROMs in daily practice, would you agree?

- Yes
- No
- No opinion

1. In what way should questionnaires be provided to patients?

- By using the electronic patient file
- By using an external program or application
- Paper version of a questionnaire
- By using an in-hospital tablet or computer
- By telephone by using interactive voice response systems
- No preference
- Other, namely (free text)

1. Which health professional should ask patients to complete questionnaires?

- Consultant specialist/health professional who is meeting the patient on the outpatient clinic
- A research nurse or research co-worker
- Automatically without any support

**Specific conditions to meet (ten questions)**

1. How long should a questionnaire take to complete?

- 1 minute
- 3 minutes
- 5 minutes
- 10 minutes
- 15 minutes
- 20 minutes
- More than 30 minutes

1. Questions must be simple enough to be understand by a twelve- year old.

- Strongly agree
- Agree
- Neither agree nor disagree
- Disagree
- Strongly disagree

1. Collected data should only be relevant for my own specialty.

- Strongly agree
- Agree
- Neither agree nor disagree
- Disagree
- Strongly disagree

1. Clinical relevant meaning of a change in score must be clear without any previous knowledge.

- Strongly agree
- Agree
- Neither agree nor disagree
- Disagree
- Strongly disagree

1. It must be evident when interference is necessary based on questionnaires scores (i.e. a deterioration of 10 points)

- Strongly agree
- Agree
- Neither agree nor disagree
- Disagree
- Strongly disagree

1. Questionnaires should always be discussed with patients despite the outcome of questionnaires.

- Strongly agree
- Agree
- Neither agree nor disagree
- Disagree
- Strongly disagree

1. Implementation of patient-reported outcomes is allowed to increase workload of health professionals as long as it is contributing to improving health care.

- Strongly agree
- Agree
- Neither agree nor disagree
- Disagree
- Strongly disagree

1. Results of questionnaires should always be capable of use in diagnostics and treatment.

- Strongly agree
- Agree
- Neither agree nor disagree
- Disagree
- Strongly disagree

1. Results of questionnaires should always be allowed to use in scientific research without interference of local ethical committees or ethical review board.

- Strongly agree
- Agree
- Neither agree nor disagree
- Disagree
- Strongly disagree

1. Patient-reported outcomes should always be part of the electronic patient file.

- Strongly agree
- Agree
- Neither agree nor disagree
- Disagree
- Strongly disagree

**Underlying construct of completion (one question)**

1. Did you complete this survey on a personal occasion or on behalf of the staff?

- On a personal occasion
- On behalf of the staff
