# Supplementary material for: Questioning the questionnaire: a Dutch national survey on generic patient-reported outcome measures in traumatology
Source: J Patient Rep Outcomes. 2025 Dec 2;9:139. doi: 10.1186/s41687-025-00969-z (PMC12672991; doi:10.1186/s41687-025-00969-z)
Supplement: Supplementary file 2 — Supplementary Material 2 [file 41687_2025_969_MOESM2_ESM.docx]

Supplemental file 3, overview of all results of the survey

| Years of experience within trauma surgery  *0-5 years*  *5-10 years*  *10-15 years*  *15-20 years*  *More than 20 years* | n= 97  24 (25%)  12 (12%)  27 (28%)  16 (17%)  18 (19%) |
| --- | --- |
| Gender  *Male*  *Female* | n= 97  83 (86%)  14 (14%) |
| Specialty  *Trauma surgeon*  *Orthopedic trauma surgeon*  *Other* | n= 97  75 (78%)  12 (12%)  10 (10%) |
| Main Affiliation  *Academic level I trauma center*  *Nonacademic level I trauma center*  *Level II hospital*  *Level III hospital* | n= 97  24 (25%)  22 (23%)  42 (43%)  9 (9%) |
| In which hospital are you currently working | Free text |
| Current position within the hospital  *Consultant specialist and staff member*  *Consultant specialist, but fellow*  *Resident*  *Other* | n= 97  80 (82%)  7 (7%)  8 (10%)  2 (2%) |
| Are patient-reported outcomes (PROMs) being provided to trauma patients within your hospital that are initiated from your department? (Questionnaires from other specialties such as the pre-operative screening department or LROI questionnaires from the orthopedic department are not included).  *Yes, routinely condition specific questionnaires*  *Yes, in selected cases condition specific*  *questionnaires*  *Yes, routinely generic questionnaires*  *Yes, in selected cases generic questionnaires*  *Yes, routinely condition specific questionnaires*  *and generic questionnaires*  *Yes, in selected cases condition specific*  *questionnaires and generic questionnaires*  *Yes, only study conditioned questionnaires*  *(condition specific/ generic)*  *No* | n= 96  3 (3.1%)  14 (14.6%)  2 (2.1%)  5 (5.2%)  6 (6.3%)  10 (10.4%)  32 (33.3%)  24 (25%) |
| At which moment are questionnaires are being provided to patients? (multiple answers can be given)  *Directly during or after presentation on the A&E*  *department*  *Before a visit of the plaster cast room*  *Before a visit of the regular outpatient clinic*  *Before an operation because of the injury*  *After an operation*  *At specifically set moments after the initial*  *trauma (i.e. after 2 weeks, 6 weeks, 3 months)*  *At specifically set moments after the operation*  *(i.e. after 2 weeks, 6 weeks, 3 months)*  *I don’t know*  *Other, namely (free text)* | n= 97  10 (10.3%)  5 (5.2%)  13 (13.4%)  2 (2.1%)  11 (11.3%)  30 (30.9%)  20 (20.6%)  12 (12.4%)  16 (16.5%) |
| In what way are questionnaires being provided to patients? (multiple answers can be given)  *By using a paper version and via a postal letter*  *A fellow worker sends the questionnaire via the*  *electronic patient file*  *A fellow worker sends the questionnaire via an*  *external program (i.e. external web viewer)*  *Fully automated by the electronic patient file*  *By telephone*  *By using a tablet in the hospital (outpatient*  *clinic or ward)*  *By using a paper version in the hospital*  *(outpatient clinic or ward)*  *I don’t know*  *Other, namely (free text)* | n= 86  11 (11.3%)  4 (4.1%)  17 (17.5%)  8 (8.2%)  10 (10.3%)  7 (7.2%)  11 (11.3%)  17 (17.5%)  12 (12.4%) |
| In what way are questionnaires being visualized, if questionnaires are being sent by using a digital method?  *Completed questionnaires are directly visible*  *within the electronic patient file*  *Completed questionnaires are available in a web*  *viewer which can be accessed by the electronic*  *patient file of the individual patient*  *Completed questionnaires are in a web viewer*  *which cannot be accessed by the electronic*  *patient file of the individual patient*  *Other, namely (free text* | n = 64  17 (26.6%)  9 (14.1%)  19 (29.7%)  19 (29.7%) |
| Which questionnaires are being used in your department? (multiple answers are possible)  *Assessment of Quality of Life (AQoL)-8D*  *European Quality Of Life-5 Dimensions*  *(EuroQol-5D)*  *Health Utility Index 2 en/of 3 (HUI2/3)*  *Hospital Anxiety And Depression Score (HADS)*  *Nottingham Health Profile (NHP)*  *PROMIS questionnaires*  *Quality of Well-Being (QWB)*  *Rosser Index*  *Sheehan Disability Scale (SDS)*  *Short Form-6 Dimensions (SF 6D)*  *Short form 12 (SF-12)*  *Short form 36 (SF-36)*  *Sickness Impact Profile*  *World Health Organization Quality Of Life*  *(WHOQOL-Bref)*  *WHO (Five) Well-Being Index (WHO-*  *15D*  *I don’t know*  *Other, namely (free text)* | n=97  3 (3.1%)  24 (24.7%)  2 (2.1%)  3 (3.1%)  0 (0%)  8 (8.2%)  0 (0%)  0 (0%)  0 (0%)  1 (1.0%)  6 (6.2%)  17 (17.5%)  0 (0%)  3 (3.1%)  1 (1%)  0 (0%)  23 (23.7%)  DASH, PRWE, Dutch Hip Fracture Audit, FFI, AOFAS, Obility score, LEFS, Rand-36. |
| Do you think generic patient-reported outcomes measures could provide useful information on your daily practice?  *Always*  *Very frequently*  *Frequently*  *Occasionally*  *Rarely*  *Never* | n=87  7 (8.0%)  14 (16.1%)  22 (25.3%)  35 (40.2%)  9 (10.3%)  0 (0%) |
| Can generic questionnaires contribute positively to the process of shared decision making in your daily practice? (i.e. in decisions about treatment options, after care or additional support)  *Always*  *Very frequently*  *Frequently*  *Occasionally*  *Rarely*  *Never* | n=87  2 (2.3%)  9 (10.3%)  27 (31%)  38 (43.7%)  11 (12.6%)  0 (0%) |
| Are you familiar with the concept of computer adaptive testing (CAT) in questionnaires?  *Yes*  *No* | n=87  39 (44.8%)  48 (55.2%) |
| CAT is based on the item-response theory. Previous questions and given answers are informing on an algorithm which selects the most suitable next question from a set of questions. Are you interested to learn more about CAT?  *Yes*  *No*  *Other, namely* | 56 (64.4%)  23 (26.4%)  8 (9.2%) |
| Is it relevant to retrieve generic physical health information about trauma patients visiting your own department? (i.e. physical function or pain interference)  *Always*  *Very frequently*  *Frequently*  *Occasionally*  *Rarely*  *Never* | n=87  20 (23%)  26 (29.9%)  36 (41.4%  4 (4.6%)  1 (1.1%)  0 (0%) |
| Is it relevant for treatment and the outcomes of treatment to be informed about social health issues of a patient? (i.e. social participation, social roles or social support)  *Always*  *Very frequently*  *Frequently*  *Occasionally*  *Rarely*  *Never* | n=86  7 (8.1%)  15 (17.4%)  36 (41.9%)  25 (29.1%)  3 (3.5%)  0 (0%) |
| Is it relevant for treatment and the outcomes of treatment to be informed about work status and performance of a patient?  *Always*  *Very frequently*  *Frequently*  *Occasionally*  *Rarely*  *Never* | n=86  2 (2.3%)  14 (16.3%)  29 (33.7%)  34 (39.5%)  6 (7.0%)  1 (1.1%) |
| Is it relevant for treatment and the outcomes of treatment to be informed about mental health issues of a patient? (i.e. anxiety, depression and *anger*)  *Always*  *Very frequently*  *Frequently*  *Occasionally*  *Rarely*  *Never* | n=86  5 (5.8%)  16 (18.6%)  38 (44.2%)  24 (27.9%)  3 (3.5%)  0 (0%) |
| Is it relevant for treatment and the outcomes of treatment to be informed about the sleeping quality of a patient?  *Always*  *Very frequently*  *Frequently*  *Occasionally*  *Rarely*  *Never* | n=86  2 (2.3%)  3 (3.5%)  19 (22.1%)  43 (50%)  17 (19.8%)  2 (2.3%) |
| Is it relevant for treatment and the outcomes of treatment to be informed about experienced fatigue of a patient?  *Always*  *Very frequently*  *Frequently*  *Occasionally*  *Rarely*  *Never* | n=86  1 (1.2%)  5 (5.8%)  20 (23.3%)  45 (52.3%)  12 (14%)  3 (3.5%) |
| *What is the most important barrier to use generic questionnaires? Please provide a maximum of three answers.*  Different interaction between health professional and patient, because of the implementation of generic questionnaires  The health professional has less control about the conversation, because of less specific information provided by the patient  Not convinced of the additional value of generic questionnaires or general health information  Some information is being provided in which the surgeon cannot directly interfere in  Privacy aspects associated with the provision of general information  Generic questionnaires might possibly take more time to discuss  Other, namely (free text) | n=97  11 (11.3%)  8 (8.2%)  25 (25.8%)  43 (44.3%)  14 (14.4%)  42 (43.3%)  23 (23.7%) |
| What will be the most important barrier to patients in completing questionnaires in general? Please provide a maximum of three answers. *Time investment*  *Privacy aspects*  *Limited computer skills*  *Low literacy*  *Visual problems*  *Insufficient insight into the additional value of questionnaires*  *Providing the patient with questionnaire(s) without discussing*  *Other, namely (free text)* | n=97  69 (71.1%)  9 (9.3%)  32 (33%)  31 (32%)  4 (4.1%)  45 (46.4%)  35 (36.1%)  4 (4.1%) |
| As a health professional, what might be the biggest advantage to use generic questionnaires? Please provide a maximum of three answers.  *The health professional gets an insight in general health aspects of the patient*  *The patient gets insight into his own general health status and the health related course*  *It is easier to compare different outcomes between subgroups when the same generic questionnaires are being used.*  *It is easier to compare the outcomes between patients connected to different specialties when the same generic questionnaires are being used.*  *The health professional is able to prepare for questions on a broader sets of health aspects*  *Outcomes can easier be used for broad scientific research purposes*  *The patient will be better informed and prepared for the outpatient clinic visit*  *Other, namely (free text)* | n=97  53 (54.6%)  25 (25.8%)  32 (33%)  11 (11.3%)  28 (28.9%)  41 (42.3%)  29 (29.9%)  3 (3.1%) |
| What should be the main goal(s) in using generic questionnaires in daily practice? Please provide a maximum of three answers.  *Efficacy measurement of interventions/treatment on health-related quality of life*  *Shared decision making based on generic information*  *Measurement and comparison of quality parameters between colleagues*  *Measurement and comparison of quality parameters between hospitals*  *Measurement of general health parameters/ general health of the patient*  *Detection of patient limitations*  *Guidance in decision making*  *To improve interaction between patient en health professional*  *Scientific research*  *Other, namely (free text)* | n=97  49 (50.5%)  31 (32%)  8 (8.2%)  11 (11.3%)  15 (15.5%)  20 (20.6%)  28 (28.9%)  19 (19.6%)  32 (33%)  1 (1%) |
| If the government, health related federation or other organization would force the use of PROMs in daily practice, would you agree?  *Yes*  *No*  *No opinion* | n=84  33 (39.3%)  41 (48.8%)  10 (11.9%) |
| In what way should questionnaires be provided to patients?  *By using the electronic patient file*  *By using an external program or application*  *Paper version of a questionnaire*  *By using an in-hospital tablet or computer*  *By telephone by using interactive voice response systems*  *No preference*  *Other, namely (free text)* | n=97  60 (61.9%)  26 (26.8%)  3 (3.1%)  22 (22.7%)  1 (1%)  5 (5.2%)  5 (5.2%) |
| Which health professional should ask patients to complete questionnaires?  *Consultant specialist/health professional who is meeting the patient on the outpatient clinic*  *A research nurse or research co-worker*  *Automatically without any support* | n=97  3 (3.1%)  42 (43.3%)  60 (61.9%) |
| How long should a questionnaire take to complete?  *1 minute*  *3 minutes*  *5 minutes*  *10 minutes*  *15 minutes*  *20 minutes*  *More than 30 minutes* | n= 84  2 (2.4%)  14 (16.7%)  39 (46.4%)  21 (25%)  6 (7.1%)  2 (2.4%)  0 (0%) |
| Questions must be simple enough to be understand by a twelve- year old.  *Strongly agree*  *Agree*  *Neither agree nor disagree*  *Disagree*  *Strongly disagree* | n=83  46 (55.4%)  32 (38.6%)  3 (3.6%  2 (2.4%)  0 (0%) |
| Collected data should only be relevant for my own specialty.  *Strongly agree*  *Agree*  *Neither agree nor disagree*  *Disagree*  *Strongly disagree* | n=83  13 (15.7%)  32 (38.6%)  24 (28.9%  13 (15.7%)  1 (1.2%) |
| Clinical relevant meaning of a change in score must be clear without any previous knowledge.  *Strongly agree*  *Agree*  *Neither agree nor disagree*  *Disagree*  *Strongly disagree* | n=83  9 (10.8%)  48 (57.8%)  19 (22.9%)  6 (7.2%)  1 (1.2%) |
| It must be evident when interference is necessary based on questionnaires scores (i.e. a deterioration of 10 points)  *Strongly agree*  *Agree*  *Neither agree nor disagree*  *Disagree*  *Strongly disagree* | n= 83  2 (2.4%)  24 (28.9%)  23 (27.7%)  24 (28.9%)  10 (12%) |
| Questionnaires should always be discussed with patients despite the outcome of questionnaires. *Strongly agree*  *Agree*  *Neither agree nor disagree*  *Disagree*  *Strongly disagree* | n= 83  4 (4.8%)  25 (30.1%)  8 (9.6%)  34 (41%)  12 (14.5%) |
| Implementation of patient-reported outcomes is allowed to increase workload of health professionals as long as it is contributing to improving health care.  *Strongly agree*  *Agree*  *Neither agree nor disagree*  *Disagree*  *Strongly disagree* | n= 83  3 (3.6%)  44 (53%)  5 (6%)  22 (26.5%)  9 (10.8%) |
| Results of questionnaires should always be capable of use in diagnostics and treatment. *Strongly agree*  *Agree*  *Neither agree nor disagree*  *Disagree*  *Strongly disagree* | n= 83  12 (14.5%)  48 (57.8%)  16 (19.3%)  5 (6%)  2 (2.4%) |
| Results of questionnaires should always be allowed to use in scientific research without interference of local ethical committees or ethical review board.  *Strongly agree*  *Agree*  *Neither agree nor disagree*  *Disagree*  *Strongly disagree* | n= 83  19 (22.9%)  33 (39.8%)  14 (16.9%)  16 (19.3%)  1 (1.2%) |
| Patient-reported outcomes should always be part of the electronic patient file.  *Strongly agree*  *Agree*  *Neither agree nor disagree*  *Disagree*  *Strongly disagree* | n= 83  30 (36.1%)  36 (43.4%)  11 (13.3%)  5 (6%)  1 (1.2%) |
| Did you complete this survey on a personal occasion or on behalf of the staff?  *On a personal occasion*  *On behalf of the staff* | n= 83  82 (98.8%)  1 (1.2%) |
